# A case–control study of maternal bathing habits and risk for birth defects in offspring

**DOI:** 10.1186/1476-069X-12-88

**Published:** 2013-10-16

**Authors:** AJ Agopian, D Kim Waller, Philip J Lupo, Mark A Canfield, Laura E Mitchell

**Affiliations:** 1 Human Genetics Center, Division of Epidemiology, Human Genetics and Environmental Sciences, University of Texas School of Public Health, 1200 Herman Pressler Dr., Houston, TX 77030, USA; 2 Division of Epidemiology, Human Genetics and Environmental Sciences, University of Texas School of Public Health, 1200 Herman Pressler Dr., Houston, TX 77030, USA; 3 Department of Pediatrics, Hematology-Oncology Section, Baylor College of Medicine, One Baylor Plaza, MS, BCM305, Houston, TX 77030, USA; 4 Birth Defects Epidemiology and Surveillance Branch, Texas Department of State Health Services, P.O. Box 149347, Austin, TX 78714-9347, USA

**Keywords:** Congenital abnormalities, Gastroschisis, Baths, Water, Hyperthermia

## Abstract

**Background:**

Nearly all women shower or take baths during early pregnancy; however, bathing habits (i.e., shower and bath length and frequency) may be related to the risk of maternal hyperthermia and exposure to water disinfection byproducts, both of which are suspected to increase risk for multiple types of birth defects. Thus, we assessed the relationships between bathing habits during pregnancy and the risk for several nonsyndromic birth defects in offspring.

**Methods:**

Data for cases with one of 13 types of birth defects and controls from the National Birth Defects Prevention Study delivered during 2000–2007 were evaluated. Logistic regression analyses were conducted separately for each type of birth defect.

**Results:**

There were few associations between shower frequency or bath frequency or length and risk for birth defects in offspring. The risk for gastroschisis in offspring was increased among women who reported showers lasting ≥15 compared to <15 minutes (adjusted odds ratio: 1.43, 95% confidence interval: 1.18-1.72). In addition, we observed modest increases in the risk for spina bifida, cleft lip with or without cleft palate, and limb reduction defects in offspring of women who showered ≥15 compared to <15 minutes. The results of comparisons among more specific categories of shower length (i.e., <15 minutes versus 15–19, 20–29, and ≥ 30 minutes) were similar.

**Conclusions:**

Our findings suggest that shower length may be associated with gastroschisis, but the modest associations with other birth defects were not supported by analyses of bath length or bath or shower frequency. Given that showering for ≥15 minutes during pregnancy is very common, further evaluation of the relationship between maternal showering habits and birth defects in offspring is worthwhile.

## Background

Approximately 76% of women in the U.S. shower at least once a day and approximately 28% of women in the U.S. take a bath at least once a day [1]. Although showering or taking baths generally improve hygiene, these activities may also result in maternal hyperthermia or exposure to water disinfection byproducts (as discussed below), and long or frequent showers or baths may result in increased exposure to either or both. However, it is unknown whether shower or bath length or frequency during early pregnancy may affect risk for birth defects.

In mice, submersion in a hot bath has been shown to induce a variety of malformations (reviewed in [2]). In epidemiologic studies, hyperthermia from heterogeneous sources (e.g., maternal fever, hot tub use, sauna use, electric blanket use) is emerging as a risk factor for miscarriage and a broad range of birth defects (reviewed in [2]). Although it has been shown that a 104 degree F hot tub can raise a woman’s core body temperature to a level similar in magnitude to those demonstrated to cause birth defects in animals (i.e., 102 degrees F) [3], the effects of bathing habits on core body temperature in humans have not been well-studied. However, short (i.e., 10 minute) warm showers and baths have been shown to result in nominal increases (up to approximately 0.5 degrees F) in rectal temperature in controlled settings [4,5]. These studies have shown linear or J-shaped increases in temperature over time, which suggests that longer showers or baths may result in more substantial increases in core temperature [5]. Previous studies have reported the average temperature of shower water in US populations to be between 101–106 degrees F [1] and typical bath temperature to be between 93–113 degrees F [6], so, for most women, longer showers or baths may result in potentially teratogenic hyperthermia (e.g., depending on length). This possibility is supported by the observation that maternal use of hot tubs during pregnancy has been associated with increased risk for a range of birth defect phenotypes in offspring [7,8].

In animal models, exposure to water disinfection byproducts (WDBPs) has induced a variety of malformations (reviewed in [9]). Showers and baths are associated with exposure to WDBPs [10,11], and epidemiological studies have inconsistently reported associations between maternal exposure via tap water and risk for birth defects (reviewed in [9,12]). Because exposure to water disinfection byproducts can occur via ingestion, dermal absorption, or inhalation of aerosolized water droplets, long or frequent showers or baths would likely increase one’s exposure to WDBPs [13]. Experimental studies in humans have shown that exposure to WDBPs from 10 minute showers or baths at home can results in higher levels of WDBP in whole blood than does drinking 1 liter of household water from the same water source [14]. However, exposure to WDBPs during showering or taking baths, specifically, and risk for birth defects has not been studied.

The purpose of this study was to assess the relationship between shower and bath length and frequency during pregnancy and the risk for several nonsyndromic birth defects in offspring, using data available from the National Birth Defects Prevention Study.

## Methods

### Study population

The National Birth Defects Prevention Study (NBDPS) is a large population-based case–control study of birth defects that includes ten surveillance sites (Arkansas, California, Georgia, Iowa, Massachusetts, New Jersey, New York, North Carolina, Texas, and Utah). The Institutional Review Boards for each site approved the study protocol and the current analyses were approved by the Institutional Review Board at the University of Texas Health Science Center.

The details of the NBDPS have been previously described [15]. Briefly, medical records for all case infants were abstracted by staff at each site and diagnoses of approximately 30 eligible defects were confirmed by NBDPS clinical geneticists based on these data [16]. To reduce heterogeneity within case groups, any case determined to have a chromosome abnormality or a single-gene disorder by the clinical geneticists was excluded from the study. For the majority of surveillance sites, cases included live births, fetal deaths (i.e., ≥20 weeks), and elective pregnancy terminations. Control infants, identified through birth certificate data or hospital birth logs, were randomly selected among all live births without birth defects in the corresponding study region.

Data on exposures before and during pregnancy, family history of birth defects, maternal conditions, and lifestyle/behavioral factors were collected during a computer-assisted interview with participating mothers of cases and controls. Questions about bath and shower habits during pregnancy were added to the interview around the year 2000. Specifically, mothers were asked how often they showered during pregnancy, as well as how many minutes were typically spent per shower. These questions were also repeated for baths. Women were also asked if they left the window open or turned on the exhaust fan when showering or taking baths.

The present analyses were conducted among subjects with estimated dates of delivery from January 1, 2000 through December 31, 2007. Cases with any of the following non-cardiac birth defect phenotypes, which have previously been associated with maternal exposure to hyperthermia or WDBPs, were included: anencephaly, spina bifida, cleft lip with or without cleft palate, cleft palate without cleft lip, microphthalmia (including anophthalmia), congenital cataract, esophageal atresia, gastroschisis, omphalocele, bilateral renal agenesis or hypoplasia, diaphragmatic hernia, limb reduction defects, and hypospadias.

### Statistical methods

Shower and bath length were categorized as <15 minutes or ≥15 minutes per occurrence, based on a median split of shower length among controls. The distributions of the following variables were compared across these two categories of shower length among controls: maternal race/ethnicity, age at delivery, education, surveillance site, body mass index, daily folic acid use (during the month before conception through the first month of pregnancy), nulliparity, smoking (during the month before pregnancy through the first trimester), season of conception, and annual household income. Data for these variables were collected during the computer-assisted telephone interview. Distributions of these variables across categories of bath length, bath frequency, and shower frequency were not compared because there was less variability in these bathing habits (i.e., the majority of women took one shower and no baths per day, see Results).

All analyses were conducted separately for each phenotype. Infants with more than one birth defect were included in analyses of each birth defect. Unconditional logistic regression was used to assess the crude relationship between shower and bath length and frequency and risk for each birth defect. Main adjusted analyses were conducted for each birth defect, adjusting for the following *a priori* potential confounders (selected based on previous literature): study site and maternal age at delivery, body mass index, education, race/ethnicity, income, parity, folic acid use, smoking, and season of conception. All analyses of hypospadias were restricted to male control infants.

Several additional analyses were conducted for shower length but not bath length, as the majority of women reported not taking baths (see Results). Main adjusted analyses of shower length were repeated again, stratified by presence of steam exhaust (use of an exhaust fan or leaving a window open during showers), because these activities are expected to decrease exposure to both hyperthermia and WDBPs. The adjusted analyses for shower length were also repeated in separate models, further adjusting for shower frequency (<1 per day, 1 per day, >1 per day), bath frequency (no baths, <1 per day, ≥1 per day), and bath length (<15 minutes, ≥15 minutes per bath). To further assess the possibility that risk may vary among the offspring of women who took longer showers, the main adjusted analyses were repeated based on more specific categories of longer shower length (i.e., 15–19, 20–29, and ≥ 30 minutes).

Because longer shower length was moderately associated with gastroschisis (see Results), and because previous literature suggests that the effects of gastroschisis risk factors may vary within specific subgroups (e.g., offspring of younger women) [17,18], additional *post-hoc* exploratory analyses where performed to evaluate the relationship between shower length and gastroschisis within specific maternal subgroups (i.e., stratified by maternal age, race/ethnicity, body mass index, and education level). The main shower length analyses were also repeated among subjects that did not take baths (i.e., those who showered exclusively).

## Results

Of 6,758 total control mothers, we observed that 95.6% (N = 6,458) reported taking showers at least once per month during pregnancy and 50.4% (N = 3,405) reported taking showers that lasted at least 15 minutes. Characteristics of control mothers were compared between women that took <15 minute and ≥15 minute showers (Table 1). Women who were Hispanic, younger, less educated, or underweight or obese, as well as women who smoked, did not use folic acid, or had lower household incomes were more likely to take ≥15 minute showers. We also tabulated the distribution of shower and bath length and frequency in control mothers (Additional file 1: Table S1). The majority (53.5%) of control mothers reported not taking baths and only 10.3% reported taking one or more baths daily.

**Table 1:** Characteristics of control mothers by shower length, National Birth Defects Prevention Study, USA, 2000-2007

**Characteristic**^**a**^	**<15 minutes**	**≥15 minutes**
Race/ethnicity		
White	2,127 (55.2)^b^	1,724 (44.8)
Black	378 (51.9)	350 (48.1)
Hispanic	495 (31.9)	1,056 (68.1)
Other	215 (44.9)	264 (55.1)
Age at delivery		
<20	190 (29.4)	457 (70.6)
20-24	604 (38.5)	966 (61.5)
25-29	891 (48.1)	960 (51.9)
30-34	971 (59.6)	658 (40.4)
35-39	488 (61.5)	306 (38.5)
≥40	85 (59.4)	58 (40.6)
Education		
<High school	390 (33.8)	763 (66.2)
High school	620 (39.6)	947 (60.4)
≥High school	2,218 (56.8)	1,684 (43.2)
Center		
Massachusetts	438 (58.5)	311 (41.5)
New Jersey	164 (49.9)	165 (50.2)
New York	295 (54.8)	243 (45.2)
Utah	300 (49.3)	309 (50.7)
Iowa	403 (56.4)	312 (43.6)
North Carolina	280 (49.1)	290 (50.9)
Arkansas	448 (51.5)	422 (48.5)
California	262 (33.6)	519 (66.5)
Texas	273 (35.0)	506 (65.0)
Atlanta	366 (52.7)	328 (47.3)
Body mass index (kg/m^2^)		
<18.5	135 (40.3)	200 (59.7)
18.5-24.9	1,763 (51.5)	1,659 (48.5)
25.0-29.9	728 (48.7)	767 (51.3)
≥30.0	507 (46.1)	593 (53.9)
Folic acid use^c^		
Yes	1,060 (60.1)	704 (39.9)
No	2,169 (44.5)	2,701 (55.5)
Nulliparity		
Yes	1,171 (44.2)	1,476 (55.8)
No	2,058 (51.6)	1,929 (48.4)
Smoking^d^		
Yes	501 (42.4)	682 (57.7)
No	2,728 (50.1)	2,722 (49.9)
Season of conception		
Summer	845 (50.2)	840 (49.9)
Fall	800 (46.8)	909 (53.2)
Winter	824 (50.3)	815 (49.7)
Spring	760 (47.5)	841 (52.5)
Annual household income		
<$10000	425 (35.6)	769 (64.4)
$10000-20000	346 (39.5)	531 (60.6)
$20000-30000	401 (46.5)	461 (53.5)
$30000-40000	313 (49.1)	325 (50.9)
$40000-50000	239 (50.7)	232 (49.3)
>$50000	1,376 (62.3)	833 (37.7)

^a^Totals do not sum to the total number of women that reported showering at least once per month due to missing data for shower length or characteristics.

^b^Row percentage.

^c^Daily use of folic acid during the month before conception through the first month of pregnancy.

^d^Any smoking during the month before pregnancy through the first trimester.

Results of the main effects of bath length (Additional file 1: Table S2) and bath and shower frequency (Additional file 1: Table S3) are presented. In crude analyses, taking ≥15 compared to <15 minute baths was associated with an increased risk of gastroschisis (OR: 1.5, 95% CI: 1.3-1.7). After adjustment for the main covariates (study site and maternal age at delivery, body mass index, education, race/ethnicity, income, parity, folic acid use, smoking, and season of conception), bath length was not significantly associated with risk for any birth defect, although there was a suggestive association with risk for gastroschisis (adjusted OR: 1.2, 95% CI: 1.0-1.4). In adjusted analyses, taking an average of 1 versus <1 shower per day was positively associated with risk for hypospadias (adjusted OR: 1.3, 95% CI: 1.0-1.6) and taking an average of >0- < 1 versus 0 baths per day was positively associated with risk for congenital cataract (adjusted OR: 1.5, 95% CI: 1.1-2.0); however, taking >1 versus 1 shower per day was negatively associated with risk for diaphragmatic hernia and anencephaly (Additional file 1: Table S3).

In crude analyses, we observed significant or borderline-significant positive associations between longer showers and 6 of 13 birth defects (Table 2). These phenotypes included anencephaly (OR: 1.4, 95% CI: 1.1-1.7), spina bifida (OR: 1.3, 95% CI: 1.1-1.5), cleft lip without cleft palate (OR: 1.3, 95% CI: 1.1-1.4), gastroschisis (OR: 2.3, 95% CI: 1.9-2.7), renal agenesis (OR: 1.5, 95% CI: 1.0-2.2), and limb reduction defects (OR: 1.2, 95% CI: 1.0-1.4). The magnitude of these associations were similar after adjustment for the main covariates (Table 2) and after further adjusting for shower frequency, bath frequency, and bath length (Additional file 1: Table S4). However, among analyses adjusted for the main covariates, which were likely underpowered for the less frequent birth defects considering the number of covariates, only the associations with gastroschisis, spina bifida, and cleft lip with or without cleft palate achieved statistical significance.

**Table 2:** Crude and adjusted odds ratios for the associations between average shower length and risk for birth defects, National Birth Defects Prevention Study, USA, 2000-2007

**Birth defect**	**N (%)**	**OR**	**95% CI**	**aOR**^**a**^	**95% CI**
Controls					
<15 minutes	3,229 (48.7)	-	-	-	-
≥15 minutes	3,405 (51.3)	-	-	-	-
Anencephaly					
<15 minutes	152 (41.3)	1.00		1.00	
≥15 minutes	216 (58.7)	1.35	1.09-1.67	1.22	0.96-1.56
Spina bifida					
<15 minutes	311 (42.8)	1.00		1.00	
≥15 minutes	416 (57.2)	1.27	1.09-1.48	1.23	1.03-1.46
Cleft lip with or without cleft palate					
<15 minutes	752 (42.5)	1.00		1.00	
≥15 minutes	1,016 (57.5)	1.27	1.14-1.41	1.14	1.01-1.28
Cleft palate without cleft lip					
<15 minutes	441 (47.8)	1.00		1.00	
≥15 minutes	481 (52.2)	1.03	0.89-1.18	1.08	0.93-1.26
Microphthalmia^b^					
<15 minutes	64 (45.4)	1.00		1.00	
≥15 minutes	77 (54.6)	1.14	0.82-1.60	0.97	0.70-1.40
Cataract					
<15 minutes	125 (50.4)	1.00		1.00	
≥15 minutes	123 (49.6)	0.93	0.72-1.20	1.02	0.77-1.34
Esophageal atresia					
<15 minutes	199 (49.5)	1.00		1.00	
≥15 minutes	203 (50.5)	0.97	0.79-1.18	1.00	0.80-1.24
Gastroschisis					
<15 minutes	235 (29.4)	1.00		1.00	
≥15 minutes	564 (70.6)	2.28	1.94-2.67	1.43	1.18-1.72
Omphalocele					
<15 minutes	115 (44.1)	1.00		1.00	
≥15 minutes	146 (55.9)	1.20	0.94-1.54	1.16	0.89-1.53
Renal agenesis^c^					
<15 minutes	40 (39.2)	1.00		1.00	
≥15 minutes	62 (60.8)	1.47	0.99-2.19	1.24	0.79-1.95
Diaphragmatic hernia					
<15 minutes	242 (47.1)	1.00		1.00	
≥15 minutes	272 (52.9)	1.07	0.89-1.28	1.07	0.88-1.31
Limb reduction defects					
<15 minutes	306 (43.9)	1.00		1.00	
≥15 minutes	391 (56.1)	1.21	1.04-1.42	1.17	0.98-1.39
Hypospadias					
<15 minutes	715 (52.6)	1.00		1.00	
≥15 minutes	645 (47.4)	0.85	0.75-0.96	1.11	0.96-1.29

Abbreviations: *OR* odds ratio, *CI* confidence interval.

^a^Adjusted for surveillance center and maternal age at delivery, body mass index, education, race/ethnicity, income, parity, folic acid use, smoking, and season of conception.

^b^Includes anopthalmia.

^c^Bilateral renal agenesis or hypoplasia.

Main adjusted analyses were repeated based on several categories of longer shower length (i.e., 15–19, 20–29, and ≥ 30 minutes) (Additional file 1: Table S5). There was little variability of effect size across these more specific categories and monotonic dose responses were not observed. Main adjusted analyses of shower length were also repeated, stratified by use of exhaust fans or leaving the window open during showers (Additional file 1: Table S6). There were few differences in the magnitude of effects between these strata. Of note, a relatively strong association was observed between risk for anencephaly and longer showers among the strata of no steam exhaust use (adjusted OR: 1.8, 95% CI: 1.1-2.7). Main analyses were also repeated among the subset of women who did not take baths (N = 3,548 control women, 52.5%), and the magnitude of the adjusted associations between shower length and anencephaly and gastroschisis were similar to the main results, but associations with spina bifida, cleft lip with or without cleft palate, renal agenesis, or limb reduction defects were not observed (Additional file 1: Table S7).

The observed association between shower length and gastroschisis varied within maternal subgroups (Table 3). For example, after adjustment for the ten main covariates, an association was observed in offspring of women who were ≥20 years at delivery (adjusted OR: 1.6, 95% CI: 1.3-2.0) but not among the offspring of younger women (adjusted OR: 1.0 95% CI: 0.7-1.5). Similarly, the association was not present among underweight women (adjusted OR: 1.0, 95% CI: 0.5-2.0) but was present among women with other body mass indexes and was not present in Hispanic women (adjusted OR: 1.1, 95% CI: 0.7-1.6), but was present in women of white, black, or other race/ethnicity. Further, the association seemed to increase in magnitude with increasing level of maternal education (e.g., women with ≥ high school education, adjusted OR: 1.8, 95% CI: 1.3, 2.5).

**Table 3:** Adjusted odds ratios for the associations between average shower length and risk for gastroschisis among maternal subgroups, National Birth Defects Prevention Study, USA, 2000-2007

**Strata**	**N (%)**	**aOR**^**a**^	**95% CI**
Maternal age			
Age <20			
<15 minutes	83 (27.0)	1.00	
≥15 minutes	225 (73.1)	1.00	0.68-1.46
Age ≥20			
<15 minutes	152 (31.0)	1.00	
≥15 minutes	339 (69.0)	1.59	1.28-1.99
Maternal Body mass index			
Underweight			
<15 minutes	19 (26.8)	1.00	
≥15 minutes	52 (73.2)	0.96	0.46-2.02
Normal weight			
<15 minutes	164 (30.4)	1.00	
≥15 minutes	376 (69.6)	1.42	1.13-1.79
Overweight or obese			
<15 minutes	47 (28.0)	1.00	
≥15 minutes	121 (72.0)	1.56	1.06-2.30
Maternal race/ethnicity			
White			
<15 minutes	137 (33.7)	1.00	
≥15 minutes	269 (66.3)	1.49	1.16-1.91
Black			
<15 minutes	25 (40.3)	1.00	
≥15 minutes	37 (59.7)	1.29	0.71-2.34
Hispanic			
<15 minutes	56 (22.0)	1.00	
≥15 minutes	199 (78.0)	1.09	0.74-1.61
Other			
<15 minutes	16 (21.3)	1.00	
≥15 minutes	59 (78.7)	1.63	0.76-3.51
Maternal education			
<High school			
<15 minutes	65 (28.0)	1.00	
≥15 minutes	167 (72.0)	1.11	0.75-1.64
High school			
<15 minutes	88 (27.8)	1.00	
≥15 minutes	299 (72.2)	1.25	0.92-1.71
>High school			
<15 minutes	81 (32.7)	1.00	
≥15 minutes	167 (67.3)	1.81	1.33-2.46

Abbreviations: *OR* odds ratio, *CI* confidence interval.

^a^Adjusted for surveillance center and maternal age, body mass index, education, race/ethnicity, income, parity, folic acid use, smoking, and season of conception; analyses stratified by body mass index, race/ethnicity, and education, were not adjusted for body mass index, race/ethnicity, and education, respectively.

## Discussion

In the first study to evaluate bathing habits during pregnancy and risk for birth defects in offspring, we observed few associations between shower frequency or bath length or frequency. However, we identified suggestive positive associations between ≥15 minute shower length and several birth defect phenotypes in offspring. Many of these associations remained in various sensitivity analyses (e.g., analyses of more specific categories of shower length). Specifically, our results suggest that longer showers may modestly increase risk for anencephaly, spina bifida, cleft lip with or without cleft palate, gastroschisis, renal agenesis, and limb reduction defects. The exact mechanism that may be responsible for these associations is unknown. Two plausible explanations include maternal exposure to water disinfection by-products and maternal hyperthermia, but it is not possible to differentiate between these mechanisms in our data and more research is needed to better understand how shower and bath length and frequency affect these exposure in humans.

One of the strongest and most consistent associations we observed was between shower length and gastroschisis. It has been shown that hyperthermic exposure can induce gastroschisis in chick embryos. For example, gastroschisis was present in 100% of 33 embryos that survived incubation at 41 degrees C (approximately 106 degrees F) and in nearly 92% of 54 that did not survive [19]. Few studies have evaluated gastroschisis and maternal hyperthermia in humans. One study has shown that maternal hot tub use during early pregnancy is associated with gastroschisis (adjusted OR:1.5, 95% CI: 1.1, 2.2) [7]. Studies evaluating gastroschisis and WDBPs have also been limited, although an association between bromoform, a water disinfection byproduct, and gastroschisis has been reported (OR:1.4, 95% CI: 1.0-1.9) [20]. Our results also support previous findings that the etiology of gastroschisis may vary between younger and older women [17,18].

Interest in understanding maternal hyperthermic exposures and birth defect risk is growing, as this mechanism is increasingly suspected to be a risk factor for a broad range of birth defects. In addition to water-related hyperthermic exposures, additional sources of interest include maternal fever, sauna use, ambient temperature, electric blankets, ultrasound and electromagnetic radiation, occupation-related hyperthermia, and medication-induced temperature increases. Although maternal hyperthermia has been evaluated relatively extensively for neural tube defects (i.e., spina bifida and anencephaly) and is strongly suspected to increase risk for neural tube defects (reviewed in [21]), hyperthermic exposures have also been associated with cleft lip with or without cleft palate, renal agenesis, and limb reduction defects (reviewed in [2,22]).

Toxicants in tap water, such as WDBPs, are also emerging as suspected risk factors for a wide range of birth defects. In addition to gastroschisis, WDBPs have been associated with neural tube defects, but do not appear to be associated with cleft lip (reviewed in [23]). Other contaminates in tap water (e.g., nitrates) have also been linked with increased risk for birth defects [24]; however, current knowledge on the relationship between maternal exposures from tap water and risk for birth defects in offspring remains limited. Because exposure to WDBPs can occur via inhalation or dermal absorption, showers represent an important potential exposure source. Other exposure sources (e.g., drinking water) are being analyzed in ongoing NBDPS studies.

Results from our evaluations of bath length and shower and bath frequency were less consistent than our main findings and did not support our shower length findings for most birth defects; however, because most women took one shower per day (68% of controls) and no baths (54% of controls), it is likely that these analyses were limited by the small amount of variability.

This study serves as a first step to understanding the relationship between shower length and birth defect risk and should be considered in light of potential limitations. Although we adjusted for study center, we were unable to directly account for variability in ambient temperature, water temperature or contaminants, maternal body temperature, or additional potential household water exposures (e.g., bathing infants, washing dishes). Furthermore, it is possible that there may have been some misclassification in our main exposure variables, as shower length was self-reported, potentially several months after pregnancy. However, showering length is somewhat habitual and may thus be relatively stable over the course of several years. Additionally, it may be difficult to measure exposure more accurately than self-report (e.g., prospectively using devices that measure temperature and water flow) in studies of rare outcomes, such as birth defects (e.g., millions of prospective subjects would be required), so these analyses based on self-reported shower length serve as an important first step. Direct measurements of maternal hyperthermia or exposure to water disinfection byproducts were unavailable, but this study has provided some rationale for follow-up studies to assess showering habits in more detail.

There were many strengths of this study, including use of data from one of the largest studies of birth defects in the world. Our use of a population-based sample that included fetal deaths and elective pregnancy terminations likely limited the influence of potential selection bias. Further, using a sample of nonsyndromic cases likely reduced case heterogeneity. We also conducted several sensitivity analyses and supplemental analyses that support our main findings.

## Conclusions

The findings reported here serve as preliminary evidence suggesting that ≥15 minute showers during pregnancy could have potential teratogenic effects, particularly for gastroschisis. Given the high prevalence of ≥15 minute showers (i.e., more than half of control women in the present study), additional studies are warranted to replicate our findings, better understand the mechanisms that may be involved, and evaluate the public health implications.

## Abbreviations

CI: Confidence interval; OR: Odds ratio; NBDPS: National birth defects prevention study; WDBPs: Water disinfection byproducts.

## Competing interests

The authors declare that they have no competing interests.

## Authors’ contributions

AJA: Conception and design of the study, analysis of the data and writing of the manuscript. LEM, PJL, MAC, DKW: Contributed to conception and design of the study, interpretation of analysis and writing of the manuscript. All five authors read and approved this version of the manuscript.

## Supplementary Material

Additional file 1: Table S1 Shower and bath characteristics among control mothers, National Birth Defects Prevention Study, USA, 2000-2007. **Table S2.** Crude and adjusted odds ratios for the associations between average bath length and risk for birth defects, National Birth Defects Prevention Study, USA, 2000-2007. **Table S3.** Crude and adjusted odds ratios for the associations between number of showers or baths per day and risk for birth defects, National Birth Defects Prevention Study, USA, 2000-2007. **Table S4.** Associations between average shower length and risk for birth defects adjusted for shower frequency and bath frequency and length, National Birth Defects Prevention Study, 2000-2007. **Table S5.** Crude and adjusted odds ratios for the associations between average shower length and risk for birth defects, National Birth Defects Prevention Study, USA, 2000-2007. **Table S6.** Adjusted odds ratios for the associations between average shower length and risk for birth defects by presence of steam exhaust, National Birth Defects Prevention Study, USA, 2000-2007. **Table S7.** Crude and adjusted odds ratios for the associations between average shower length and risk for birth defects among participants who did not take baths, National Birth Defects Prevention Study, USA, 2000-2007. Click here for file
