# Supplementary material for: A case–control study of maternal bathing habits and risk for birth defects in offspring
Source: Environ Health. 2013 Oct 16;12:88. doi: 10.1186/1476-069X-12-88 (PMC4015781; doi:10.1186/1476-069X-12-88)
Supplement: Additional file 1: Table S1 — Shower and bath characteristics among control mothers, National Birth Defects Prevention Study, USA, 2000-2007. Table S2. Crude and adjusted odds ratios for the associations between average bath length and risk for birth defects, National Birth Defects Prevention Study, USA, 2000-2007. Table S3. Crude and adjusted odds ratios for the associations between number of showers or baths per day and risk for birth defects, National Birth Defects Prevention Study, USA, 2000-2007. Table S4. Associations between average shower length and risk for birth defects adjusted for shower frequency and bath frequency and length, National Birth Defects Prevention Study, 2000-2007. Table S5. Crude and adjusted odds ratios for the associations between average shower length and risk for birth defects, National Birth Defects Prevention Study, USA, 2000-2007. Table S6. Adjusted odds ratios for the associations between average shower length and risk for birth defects by presence of steam exhaust, National Birth Defects Prevention Study, USA, 2000-2007. Table S7. Crude and adjusted odds ratios for the associations between average shower length and risk for birth defects among participants who did not take baths, National Birth Defects Prevention Study, USA, 2000-2007. [file 1476-069X-12-88-S1.doc]

Supplemental Table 1. Shower and bath characteristics among control mothers, National Birth Defects Prevention Study, USA, 2000-2007

|  | Shower | | Bath | |
| --- | --- | --- | --- | --- |
| Characteristic | Length  (minutes) | Frequency  (per week) | Length  (minutes) | Frequency  (per week) |
| Mean | 14.4 | 7.9 | 11.4 | 1.4 |
| 1^st^ percentile | 0 | 0 | 0 | 0 |
| 25^th^ percentile | 10 | 7 | 0 | 0 |
| Median | 15 | 7 | 0 | 0 |
| 75^th^ percentile | 20 | 7 | 20 | 1 |
| 99^th^ percentile | 45 | 21 | 60 | 14 |

Supplemental Table 2. Crude and adjusted odds ratios for the associations between average bath length and risk for birth defects, National Birth Defects Prevention Study, USA, 2000-2007

| Birth Defect | N (%) | OR | 95% CI | aOR^a^ | 95% CI |
| --- | --- | --- | --- | --- | --- |
| Controls |  |  |  |  |  |
| <15 minutes | 4,599 (69.3) | - | - | - | - |
| ≥15 minutes | 2,034 (30.7) | - | - | - | - |
| Anencephaly |  |  |  |  |  |
| <15 minutes | 266 (72.3) | 1.00 |  | 1.00 |  |
| ≥15 minutes | 102 (27.7) | 0.87 | 0.69-1.10 | 0.93 | 0.72-1.21 |
| Spina bifida |  |  |  |  |  |
| <15 minutes | 513 (70.7) | 1.00 |  | 1.00 |  |
| ≥15 minutes | 213 (29.3) | 0.94 | 0.79-1.11 | 0.97 | 0.80-1.16 |
| Cleft lip with or without cleft palate |  |  |  |  |  |
| <15 minutes | 1,237 (70.0) | 1.00 |  | 1.00 |  |
| ≥15 minutes | 530 (30.0) | 0.98 | 0.87-1.10 | 1.02 | 0.90-1.15 |
| Cleft palate without cleft lip |  |  |  |  |  |
| <15 minutes | 638 (69.2) | 1.00 |  | 1.00 |  |
| ≥15 minutes | 284 (30.8) | 1.02 | 0.87-1.18 | 1.08 | 0.92-1.27 |
| Microphthalmia^c^ |  |  |  |  |  |
| <15 minutes | 98 (69.5) | 1.00 |  | 1.00 |  |
| ≥15 minutes | 43 (30.5) | 0.99 | 0.69-1.43 | 0.89 | 0.60-1.32 |
| Cataract |  |  |  |  |  |
| <15 minutes | 163 (65.7) | 1.00 |  | 1.00 |  |
| ≥15 minutes | 85 (34.3) | 1.18 | 0.90-1.54 | 1.21 | 0.91-1.61 |
| Esophageal atresia |  |  |  |  |  |
| <15 minutes | 291 (72.4) | 1.00 |  | 1.00 |  |
| ≥15 minutes | 111 (27.6) | 0.86 | 0.69-1.08 | 1.02 | 0.80-1.31 |
| Gastroschisis |  |  |  |  |  |
| <15 minutes | 481 (60.2) | 1.00 |  | 1.00 |  |
| ≥15 minutes | 318 (39.8) | 1.50 | 1.29-1.74 | 1.17 | 0.98-1.40 |
| Omphalocele |  |  |  |  |  |
| <15 minutes | 181 (69.1) | 1.00 |  | 1.00 |  |
| ≥15 minutes | 81 (30.9) | 1.01 | 0.78-1.32 | 0.99 | 0.75-1.33 |
| Renal agenesis^d^ |  |  |  |  |  |
| <15 minutes | 71 (69.6) | 1.00 |  | 1.00 |  |
| ≥15 minutes | 31 (30.4) | 0.99 | 0.65-1.51 | 0.88 | 0.55-1.41 |
| Diaphragmatic hernia |  |  |  |  |  |
| <15 minutes | 360 (70.0) | 1.00 |  | 1.00 |  |
| ≥15 minutes | 154 (30.0) | 0.97 | 0.80-1.18 | 1.01 | 0.82-1.25 |
| Limb reduction defects |  |  |  |  |  |
| <15 minutes | 486 (69.7) | 1.00 |  | 1.00 |  |
| ≥15 minutes | 211 (30.3) | 0.98 | 0.83-1.16 | 1.04 | 0.87-1.26 |
| Hypospadias |  |  |  |  |  |
| <15 minutes | 977 (71.9) | 1.00 |  | 1.00 |  |
| ≥15 minutes | 382 (28.1) | 0.86 | 0.75-0.99 | 0.94 | 0.80-1.10 |

Abbreviations: OR, odds ratio; CI, confidence interval

^a^ Adjusted for surveillance center and maternal age at delivery, body mass index, education, race/ethnicity, income, parity, folic acid use, smoking, and season of conception

^c^ Includes anopthalmia

^d^ Bilateral renal agenesis or hypoplasia

Supplemental Table 3. Crude and adjusted odds ratios for the associations between number of showers or baths per day and risk for birth defects, National Birth Defects Prevention Study, USA, 2000-2007

| Birth Defect |  |  | Showers |  |  |  |  |  |  | Baths |  |  |
| --- | --- | --- | --- | --- | --- | --- | --- | --- | --- | --- | --- | --- |
|  | N (%) | OR | 95% CI | aOR^a^ | 95% CI |  | Frequency | N (%) | OR | 95% CI | aOR^b^ | 95% CI |
| Controls |  |  |  |  |  |  |  |  |  |  |  |  |
| <1 per day | 857 (12.9) | - | - | - | - |  | No baths | 3,548 (53.5) | - | - | - | - |
| 1 per day | 4,537 (68.3) | - | - | - | - |  | <1 per day | 2,405 (36.3) | - | - | - | - |
| >1 per day | 1,245 (18.8) | - | - | - | - |  | ≥1 per day | 682 (10.3) | - | - | - | - |
| Anencephaly |  |  |  |  |  |  |  |  |  |  |  |  |
| <1 per day | 49 (13.3) | 0.99 | 0.72-1.36 | 1.09 | 0.78-1.53 |  | No baths | 202 (54.9) | 1.02 | 0.82-1.28 | 0.98 | 0.76-1.27 |
| 1 per day | 262 (71.2) | 1.00 |  | 1.00 |  |  | <1 per day | 134 (36.4) | 1.00 |  | 1.00 |  |
| >1 per day | 57 (15.5) | 0.79 | 0.59-1.06 | 0.61 | 0.43-0.87 |  | ≥1 per day | 32 (8.7) | 0.84 | 0.57-1.25 | 0.86 | 0.56-1.33 |
| Spina bifida |  |  |  |  |  |  |  |  |  |  |  |  |
| <1 per day | 85 (11.7) | 0.87 | 0.68-1.11 | 0.92 | 0.71-1.19 |  | No baths | 409 (56.3) | 1.12 | 0.95-1.32 | 1.06 | 0.88-1.30 |
| 1 per day | 517 (71.0) | 1.00 |  | 1.00 |  |  | <1 per day | 248 (34.1) | 1.00 |  | 1.00 |  |
| >1 per day | 126 (17.3) | 0.89 | 0.72-1.09 | 0.83 | 0.65-1.06 |  | ≥1 per day | 70 (9.6) | 1.00 | 0.75-1.32 | 0.98 | 0.72-1.34 |
| Cleft lip with or without cleft palate | |  |  |  |  |  |  |  |  |  |  |  |
| <1 per day | 236 (13.4) | 1.04 | 0.89-1.22 | 1.06 | 0.89-1.25 |  | No baths | 971 (55.0) | 1.04 | 0.93-1.17 | 1.00 | 0.88-1.14 |
| 1 per day | 1,225 (69.3) | 1.00 |  | 1.00 |  |  | <1 per day | 619 (35.0) | 1.00 |  | 1.00 |  |
| >1 per day | 307 (17.4) | 0.90 | 0.78-1.04 | 0.94 | 0.80-1.10 |  | ≥1 per day | 177 (10.0) | 0.99 | 0.82-1.20 | 1.03 | 0.84-1.26 |
| Cleft palate without cleft lip | |  |  |  |  |  |  |  |  |  |  |  |
| <1 per day | 125 (13.6) | 1.03 | 0.84-1.27 | 1.02 | 0.82-1.27 |  | No baths | 499 (54.1) | 0.99 | 0.86-1.15 | 0.93 | 0.79-1.10 |
| 1 per day | 653 (70.8) | 1.00 |  | 1.00 |  |  | <1 per day | 335 (36.3) | 1.00 |  | 1.00 |  |
| >1 per day | 144 (15.6) | 0.79 | 0.65-0.96 | 0.82 | 0.66-1.02 |  | ≥1 per day | 88 (9.5) | 0.91 | 0.71-1.17 | 0.96 | 0.74-1.26 |
| Microphthalmia^b^ |  |  |  |  |  |  |  |  |  |  |  |  |
| <1 per day | 21 (14.9) | 1.22 | 0.76-1.98 | 1.18 | 0.71-1.97 |  | No baths | 75 (53.2) | 1.08 | 0.75-1.56 | 1.11 | 0.74-1.66 |
| 1 per day | 91 (64.5) | 1.00 |  | 1.00 |  |  | <1 per day | 47 (33.3) | 1.00 |  | 1.00 |  |
| >1 per day | 29 (20.6) | 1.16 | 0.76-1.78 | 1.02 | 0.63-1.65 |  | ≥1 per day | 19 (13.5) | 1.43 | 0.83-2.45 | 1.20 | 0.70-2.15 |
| Cataract |  |  |  |  |  |  |  |  |  |  |  |  |
| <1 per day | 26 (10.4) | 0.76 | 0.50-1.16 | 0.78 | 0.51-1.20 |  | No baths | 111 (44.8) | 0.69 | 0.52-0.90 | 0.67 | 0.50-0.89 |
| 1 per day | 181 (72.7) | 1.00 |  | 1.00 |  |  | <1 per day | 110 (44.4) | 1.00 |  | 1.00 |  |
| >1 per day | 42 (16.9) | 0.85 | 0.60-1.20 | 0.93 | 0.64-1.37 |  | ≥1 per day | 27 (10.9) | 0.86 | 0.56-1.33 | 0.79 | 0.50-1.26 |
| Esophageal atresia |  |  |  |  |  |  |  |  |  |  |  |  |
| <1 per day | 43 (10.7) | 0.76 | 0.55-1.06 | 0.88 | 0.62-1.24 |  | No baths | 231 (57.3) | 1.07 | 0.86-1.32 | 0.91 | 0.72-1.14 |
| 1 per day | 299 (74.2) | 1.00 |  | 1.00 |  |  | <1 per day | 147 (36.5) | 1.00 |  | 1.00 |  |
| >1 per day | 61 (15.1) | 0.74 | 0.56-0.99 | 0.95 | 0.69-1.30 |  | ≥1 per day | 25 (6.2) | 0.60 | 0.39-0.92 | 0.68 | 0.43-1.08 |
| Gastroschisis |  |  |  |  |  |  |  |  |  |  |  |  |
| <1 per day | 80 (10.0) | 0.78 | 0.61-1.00 | 0.76 | 0.57-1.01 |  | No baths | 372 (46.6) | 0.77 | 0.66-0.90 | 0.96 | 0.80-1.17 |
| 1 per day | 541 (67.7) | 1.00 |  | 1.00 |  |  | <1 per day | 328 (41.1) | 1.00 |  | 1.00 |  |
| >1 per day | 178 (22.3) | 1.20 | 1.00-1.44 | 0.89 | 0.71-1.11 |  | ≥1 per day | 99 (12.4) | 1.06 | 0.84-1.35 | 0.96 | 0.72-1.27 |
| Omphalocele |  |  |  |  |  |  |  |  |  |  |  |  |
| <1 per day | 29 (11.1) | 0.87 | 0.58-1.29 | 0.84 | 0.55-1.28 |  | No baths | 140 (53.4) | 0.99 | 0.76-1.29 | 1.03 | 0.77-1.38 |
| 1 per day | 177 (67.6) | 1.00 |  | 1.00 |  |  | <1 per day | 96 (36.6) | 1.00 |  | 1.00 |  |
| >1 per day | 56 (21.4) | 1.15 | 0.85-1.57 | 1.00 | 0.70-1.42 |  | ≥1 per day | 26 (9.9) | 0.96 | 0.61-1.49 | 0.94 | 0.58-1.50 |
| Renal agenesis^c^ |  |  |  |  |  |  |  |  |  |  |  |  |
| <1 per day | 14 (13.6) | 1.09 | 0.61-1.95 | 0.97 | 0.51-1.85 |  | No baths | 56 (54.9) | 1.15 | 0.75-1.77 | 1.25 | 0.76-2.04 |
| 1 per day | 68 (66.0) | 1.00 |  | 1.00 |  |  | <1 per day | 33 (32.4) | 1.00 |  | 1.00 |  |
| >1 per day | 21 (20.4) | 1.13 | 0.69-1.84 | 0.85 | 0.48-1.53 |  | ≥1 per day | 13 (12.8) | 1.39 | 0.73-2.66 | 1.48 | 0.75-2.94 |
| Diaphragmatic hernia |  |  |  |  |  |  |  |  |  |  |  |  |
| <1 per day | 63 (12.3) | 0.88 | 0.67-1.16 | 0.85 | 0.63-1.15 |  | No baths | 279 (54.3) | 0.98 | 0.81-1.19 | 0.96 | 0.78-1.18 |
| 1 per day | 380 (73.9) | 1.00 |  | 1.00 |  |  | <1 per day | 193 (37.6) | 1.00 |  | 1.00 |  |
| >1 per day | 71 (13.8) | 0.68 | 0.52-0.88 | 0.67 | 0.50-0.91 |  | ≥1 per day | 42 (8.2) | 0.77 | 0.54-1.08 | 0.76 | 0.52-1.11 |
| Limb reduction defects |  |  |  |  |  |  |  |  |  |  |  |  |
| <1 per day | 75 (10.8) | 0.80 | 0.62-1.04 | 0.87 | 0.67-1.14 |  | No baths | 381 (54.7) | 1.09 | 0.92-1.29 | 1.18 | 0.95-1.47 |
| 1 per day | 494 (70.9) | 1.00 |  | 1.00 |  |  | <1 per day | 238 (34.2) | 1.00 |  | 1.00 |  |
| >1 per day | 128 (18.4) | 0.94 | 0.77-1.16 | 0.92 | 0.73-1.16 |  | ≥1 per day | 78 (11.2) | 1.16 | 0.88-1.51 | 1.00 | 0.72-1.39 |
| Hypospadias |  |  |  |  |  |  |  |  |  |  |  |  |
| <1 per day | 133 (9.8) | 0.73 | 0.59-0.90 | 0.79 | 0.63-0.99 |  | No baths | 748 (55.1) | 1.02 | 0.89-1.17 | 0.98 | 0.84-1.15 |
| 1 per day | 1,042 (76.7) | 1.00 |  | 1.00 |  |  | <1 per day | 503 (37.0) | 1.00 |  | 1.00 |  |
| >1 per day | 184 (13.5) | 0.65 | 0.54-0.78 | 0.88 | 0.71-1.10 |  | ≥1 per day | 107 (7.9) | 0.71 | 0.56-0.90 | 0.81 | 0.62-1.06 |

Abbreviations: OR, odds ratio; CI, confidence interval

^a^ Adjusted for surveillance center and maternal age at delivery, body mass index, education, race/ethnicity, income, parity, folic acid use, smoking, and season of conception

^b^ Includes anopthalmia

^c^Bilateral renal agenesis or hypoplasia

Supplemental Table 4. Associations between average shower length and risk for birth defects adjusted for shower frequency and bath frequency and length, National Birth Defects Prevention Study, 2000-2007

| Birth Defect | aOR^a^ | 95% CI |
| --- | --- | --- |
| Anencephaly |  |  |
| <15 minutes | 1.00 |  |
| ≥15 minutes | 1.25 | 0.97-1.61 |
| Spina bifida |  |  |
| <15 minutes | 1.00 |  |
| ≥15 minutes | 1.47 | 0.99-2.18 |
| Cleft lip with or without cleft palate |  |  |
| <15 minutes | 1.00 |  |
| ≥15 minutes | 1.14 | 1.01-1.28 |
| Cleft palate without cleft lip |  |  |
| <15 minutes | 1.00 |  |
| ≥15 minutes | 1.08 | 0.92-1.26 |
| Microphthalmia^b^ |  |  |
| <15 minutes | 1.00 |  |
| ≥15 minutes | 0.99 | 0.68-1.44 |
| Cataract |  |  |
| <15 minutes | 1.00 |  |
| ≥15 minutes | 1.05 | 0.79-1.39 |
| Esophageal atresia |  |  |
| <15 minutes | 1.00 |  |
| ≥15 minutes | 0.99 | 0.79-1.25 |
| Gastroschisis |  |  |
| <15 minutes | 1.00 |  |
| ≥15 minutes | 1.37 | 1.13-1.67 |
| Omphalocele |  |  |
| <15 minutes | 1.00 |  |
| ≥15 minutes | 1.16 | 0.88-1.53 |
| Renal agenesis^c^ |  |  |
| <15 minutes | 1.00 |  |
| ≥15 minutes | 1.28 | 0.80-2.04 |
| Diaphragmatic hernia |  |  |
| <15 minutes | 1.00 |  |
| ≥15 minutes | 1.07 | 0.87-1.30 |
| Limb reduction defects |  |  |
| <15 minutes | 1.00 |  |
| ≥15 minutes | 1.17 | 0.98-1.40 |
| Hypospadias |  |  |
| <15 minutes | 1.00 |  |
| ≥15 minutes | 1.13 | 0.98-1.32 |

^a^ Adjusted for number of showers per day, number of baths per day, average bath length, surveillance center and maternal age at delivery, body mass index, education, race/ethnicity, income, parity, folic acid use, smoking, and season of conception

^b^ Includes anopthalmia

^c^ Bilateral renal agenesis or hypoplasia

Supplemental Table 5. Crude and adjusted odds ratios for the associations between average shower length and risk for birth defects, National Birth Defects Prevention Study, USA, 2000-2007

| Birth Defect | N (%) | OR | 95% CI | aOR^a^ | 95% CI |
| --- | --- | --- | --- | --- | --- |
| Controls |  |  |  |  |  |
| <15 minutes | 3,229 (48.7) | - | - | - | - |
| 15-19 minutes | 1,694 (25.5) | - | - | - | - |
| 20-29 minutes | 1,106 (16.7) | - | - | - | - |
| ≥30 minutes | 605 (9.1) | - | - | - | - |
| Anencephaly |  |  |  |  |  |
| <15 minutes | 152 (41.3) | 1.00 |  | 1.00 |  |
| 15-19 minutes | 100 (27.2) | 1.25 | 0.97-1.63 | 1.27 | 0.96-1.69 |
| 20-29 minutes | 74 (20.1) | 1.42 | 1.07-1.89 | 1.11 | 0.80-1.56 |
| ≥30 minutes | 42 (11.4) | 1.48 | 1.04-2.10 | 1.29 | 0.85-1.96 |
| Spina bifida |  |  |  |  |  |
| <15 minutes | 311 (42.8) | 1.00 |  | 1.00 |  |
| 15-19 minutes | 206 (28.3) | 1.26 | 1.05-1.52 | 1.27 | 1.04-1.55 |
| 20-29 minutes | 135 (18.6) | 1.27 | 1.02-1.57 | 1.19 | 0.94-1.51 |
| ≥30 minutes | 75 (10.3) | 1.29 | 0.99-1.68 | 1.14 | 0.83-1.58 |
| Cleft lip with or without cleft palate |  |  |  |  |  |
| <15 minutes | 752 (42.5) | 1.00 |  | 1.00 |  |
| 15-19 minutes | 498 (28.2) | 1.26 | 1.10-1.43 | 1.16 | 1.01-1.33 |
| 20-29 minutes | 325 (18.4) | 1.25 | 1.08-1.44 | 1.09 | 0.93-1.28 |
| ≥30 minutes | 193 (10.9) | 1.36 | 1.13-1.63 | 1.15 | 0.93-1.42 |
| Cleft palate without cleft lip |  |  |  |  |  |
| <15 minutes | 441 (47.8) | 1.00 |  | 1.00 |  |
| 15-19 minutes | 236 (25.6) | 1.01 | 0.86-1.20 | 1.07 | 0.89-1.28 |
| 20-29 minutes | 171 (18.6) | 1.12 | 0.92-1.35 | 1.15 | 0.94-1.41 |
| ≥30 minutes | 74 (8.0) | 0.89 | 0.68-1.15 | 0.98 | 0.73-1.32 |
| Microphthalmia^b^ |  |  |  |  |  |
| <15 minutes | 64 (45.4) | 1.00 |  | 1.00 |  |
| 15-19 minutes | 42 (29.8) | 1.25 | 0.84-1.85 | 1.08 | 0.71-1.66 |
| 20-29 minutes | 27 (19.2) | 1.23 | 0.78-1.94 | 0.99 | 0.60-1.64 |
| ≥30 minutes | 8 (1.3) | -^c^ | - | -^c^ | - |
| Cataract |  |  |  |  |  |
| <15 minutes | 125 (50.4) | 1.00 |  | 1.00 |  |
| 15-19 minutes | 78 (1.14) | 1.19 | 0.89-1.59 | 1.26 | 0.93-1.70 |
| 20-29 minutes | 36 (14.5) | 0.84 | 0.58-1.23 | 0.84 | 0.55-1.27 |
| ≥30 minutes | 9 (3.6) | -^c^ | - | -^c^ | - |
| Esophageal atresia |  |  |  |  |  |
| <15 minutes | 199 (49.5) | 1.00 |  | 1.00 |  |
| 15-19 minutes | 105 (26.1) | 1.01 | 0.79-1.28 | 0.96 | 0.73-1.24 |
| 20-29 minutes | 65 (16.2) | 0.95 | 0.72-1.27 | 1.02 | 0.74-1.39 |
| ≥30 minutes | 33 (5.2) | 0.89 | 0.61-1.29 | 1.14 | 0.73-1.76 |
| Gastroschisis |  |  |  |  |  |
| <15 minutes | 235 (29.4) | 1.00 |  | 1.00 |  |
| 15-19 minutes | 236 (29.5) | 1.91 | 1.58-2.32 | 1.47 | 1.18-1.83 |
| 20-29 minutes | 181 (22.7) | 2.25 | 1.83-2.76 | 1.45 | 1.14-1.84 |
| ≥30 minutes | 147 (18.4) | 3.34 | 2.67-4.18 | 1.30 | 0.99-1.72 |
| Omphalocele |  |  |  |  |  |
| <15 minutes | 115 (44.1) | 1.00 |  | 1.00 |  |
| 15-19 minutes | 77 (29.5) | 1.28 | 0.95-1.71 | 1.27 | 0.93-1.73 |
| 20-29 minutes | 39 (14.9) | 0.99 | 0.68-1.43 | 0.88 | 0.59-1.33 |
| ≥30 minutes | 30 (11.5) | 1.39 | 0.92-2.10 | 1.41 | 0.88-2.25 |
| Renal agenesis^d^ |  |  |  |  |  |
| <15 minutes | 40 (39.2) | 1.00 |  | 1.00 |  |
| 15-19 minutes | 28 (27.5) | 1.33 | 0.82-2.17 | 1.27 | 0.75-2.15 |
| 20-29 minutes | 20 (19.6) | 1.46 | 0.85-2.51 | 1.08 | 0.57-2.02 |
| ≥30 minutes | 14 (13.7) | 1.87 | 1.01-3.45 | 1.49 | 0.74-3.01 |
| Diaphragmatic hernia |  |  |  |  |  |
| <15 minutes | 242 (47.1) | 1.00 |  | 1.00 |  |
| 15-19 minutes | 149 (29.0) | 1.17 | 0.95-1.45 | 1.19 | 0.95-1.49 |
| 20-29 minutes | 79 (15.4) | 0.95 | 0.73-1.24 | 0.89 | 0.66-1.19 |
| ≥30 minutes | 44 (6.8) | 0.97 | 0.70-1.35 | 1.05 | 0.72-1.53 |
| Limb reduction defects |  |  |  |  |  |
| <15 minutes | 306 (43.9) | 1.00 |  | 1.00 |  |
| 15-19 minutes | 206 (29.6) | 1.28 | 1.07-1.55 | 1.23 | 1.00-1.50 |
| 20-29 minutes | 125 (17.9) | 1.19 | 0.96-1.49 | 1.16 | 0.91-1.47 |
| ≥30 minutes | 60 (8.6) | 1.05 | 0.78-1.40 | 0.99 | 0.71-1.37 |
| Hypospadias |  |  |  |  |  |
| <15 minutes | 715 (52.6) | 1.00 |  | 1.00 |  |
| 15-19 minutes | 360 (26.5) | 0.95 | 0.82-1.10 | 1.09 | 0.92-1.29 |
| 20-29 minutes | 212 (15.6) | 0.86 | 0.72-1.03 | 1.18 | 0.95-1.45 |
| ≥30 minutes | 73 (5.4) | 0.54 | 0.41-0.70 | 1.08 | 0.78-1.49 |

Abbreviations: OR, odds ratio; CI, confidence interval

^a^ Adjusted for surveillance center and maternal age, body mass index, education, race/ethnicity, income, parity, folic acid use, smoking, and season of conception

^b^ Includes anopthalmia

^c^ Not computed due to small numbers

^d^ Bilateral renal agenesis or hypoplasia

Supplemental Table 6. Adjusted odds ratios for the associations between average shower length and risk for birth defects by presence of steam exhaust, National Birth Defects Prevention Study, USA, 2000-2007

| Birth Defect | Steam Exhaust^a^ | | | No Steam Exhaust^a^ | | |
| --- | --- | --- | --- | --- | --- | --- |
|  | N (%) | aOR^b^ | 95% CI | N (%) | aOR^b^ | 95% CI |
| Controls |  |  |  |  |  |  |
| <15 minutes | 1,268 (50.0) | - | - | 1,102 (49.4) | - | - |
| ≥15 minutes | 1,270 (50.0) | - | - | 1,130 (50.6) | - | - |
| Anencephaly |  |  |  |  |  |  |
| <15 minutes | 52 (36.6) | 1.00 |  | 45 (36.3) | 1.00 |  |
| ≥15 minutes | 90 (63.4) | 1.47 | 0.98-2.20 | 79 (63.7) | 1.76 | 1.14-2.71 |
| Spina bifida |  |  |  |  |  |  |
| <15 minutes | 116 (43.1) | 1.00 |  | 102 (41.3) | 1.00 |  |
| ≥15 minutes | 153 (56.9) | 1.20 | 0.89-1.61 | 145 (58.7) | 1.30 | 0.96-1.76 |
| Cleft lip with or without cleft palate |  |  |  |  |  |  |
| <15 minutes | 277 (41.6) | 1.00 |  | 263 (43.3) | 1.00 |  |
| ≥15 minutes | 389 (58.4) | 1.29 | 1.06-1.56 | 345 (56.7) | 1.10 | 0.90-1.35 |
| Cleft palate without cleft lip |  |  |  |  |  |  |
| <15 minutes | 166 (47.2) | 1.00 |  | 142 (47.5) | 1.00 |  |
| ≥15 minutes | 186 (52.8) | 1.24 | 0.97-1.59 | 157 (52.5) | 1.19 | 0.91-1.56 |
| Microphthalmia^c^ |  |  |  |  |  |  |
| <15 minutes | 30 (49.2) | 1.00 |  | 19 (40.4) | 1.00 |  |
| ≥15 minutes | 31 (50.8) | 0.93 | 0.52-1.66 | 28 (59.6) | 1.25 | 0.66-2.38 |
| Cataract |  |  |  |  |  |  |
| <15 minutes | 41 (44.6) | 1.00 |  | 48 (52.8) | 1.00 |  |
| ≥15 minutes | 51 (55.4) | 1.36 | 0.86-2.14 | 43 (47.3) | 0.89 | 0.55-1.41 |
| Esophageal atresia |  |  |  |  |  |  |
| <15 minutes | 199 (49.5) | 1.00 |  |  | 1.00 |  |
| ≥15 minutes | 203 (50.5) | 0.97 | 0.79-1.18 |  | 1.00 | 0.80-1.24 |
| Gastroschisis |  |  |  |  |  |  |
| <15 minutes | 79 (28.4) | 1.00 |  | 84 (31.0) | 1.00 |  |
| ≥15 minutes | 199 (36.3) | 1.40 | 1.02-1.94 | 187 (69.0) | 1.37 | 0.99-1.89 |
| Omphalocele |  |  |  |  |  |  |
| <15 minutes | 49 (46.7) | 1.00 |  | 41 (44.1) | 1.00 |  |
| ≥15 minutes | 56 (53.3) | 1.16 | 0.75-1.80 | 52 (55.9) | 1.12 | 0.70-1.79 |
| Renal agenesis^d^ |  |  |  |  |  |  |
| <15 minutes | 16 (31.4) | 1.00 |  | 17 (46.0) | 1.00 |  |
| ≥15 minutes | 35 (68.6) | -^e^ | - | 20 (54.1) | -^e^ | - |
| Diaphragmatic hernia |  |  |  |  |  |  |
| <15 minutes | 85 (45.2) | 1.00 |  | 84 (44.7) | 1.00 |  |
| ≥15 minutes | 103 (54.8) | 1.36 | 0.97-1.89 | 104 (55.3) | 1.27 | 0.90-1.77 |
| Limb reduction defects |  |  |  |  |  |  |
| <15 minutes | 116 (43.0) | 1.00 |  | 105 (44.9) | 1.00 |  |
| ≥15 minutes | 154 (57.0) | 1.32 | 0.99-1.75 | 129 (55.1) | 1.11 | 0.82-1.50 |
| Hypospadias |  |  |  |  |  |  |
| <15 minutes | 301 (52.7) | 1.00 |  | 227 (53.3) | 1.00 |  |
| ≥15 minutes | 270 (47.3) | 1.28 | 1.02-1.62 | 199 (46.7) | 1.07 | 0.82-1.39 |

Abbreviations: OR, odds ratio; CI, confidence interval

^a^ Use of an exhaust fan or leaving a window open during showers

^b^ Adjusted for surveillance center and maternal age at delivery, body mass index, education, race/ethnicity, income, parity, folic acid use, smoking, and season of conception

^c^ Includes anopthalmia

^d^ Bilateral renal agenesis or hypoplasia

^e^ Not calculated due to small numbers

Supplemental Table 7. Crude and adjusted odds ratios for the associations between average shower length and risk for birth defects among participants who did not take baths, National Birth Defects Prevention Study, USA, 2000-2007

| Birth Defect | N (%) | OR | 95% CI | aOR^a^ | 95% CI |
| --- | --- | --- | --- | --- | --- |
| Controls |  |  |  |  |  |
| <15 minutes | 1,693 (47.8) | - | - | - | - |
| ≥15 minutes | 1,850 (52.2) | - | - | - | - |
| Anencephaly |  |  |  |  |  |
| <15 minutes | 79 (39.3) | 1.00 |  | 1.00 |  |
| ≥15 minutes | 122 (60.7) | 1.41 | 1.06-1.89 | 1.27 | 0.91-1.79 |
| Spina bifida |  |  |  |  |  |
| <15 minutes | 181 (44.4) | 1.00 |  | 1.00 |  |
| ≥15 minutes | 227 (55.6) | 1.15 | 0.93-1.41 | 1.10 | 0.87-1.40 |
| Cleft lip with or without cleft palate |  |  |  |  |  |
| <15 minutes | 406 (41.8) | 1.00 |  | 1.00 |  |
| ≥15 minutes | 566 (58.2) | 1.27 | 1.10-1.47 | 1.13 | 0.96-1.32 |
| Cleft palate without cleft lip |  |  |  |  |  |
| <15 minutes | 248 (50.0) | 1.00 |  | 1.00 |  |
| ≥15 minutes | 248 (50.0) | 0.91 | 0.76-1.10 | 1.01 | 0.82-1.25 |
| Microphthalmia^c^ |  |  |  |  |  |
| <15 minutes | 36 (48.0) | 1.00 |  | 1.00 |  |
| ≥15 minutes | 39 (52.0) | 0.99 | 0.63-1.57 | 0.73 | 0.44-1.23 |
| Cataract |  |  |  |  |  |
| <15 minutes | 58 (51.8) | 1.00 |  | 1.00 |  |
| ≥15 minutes | 54 (48.2) | 0.85 | 0.58-1.24 | 0.82 | 0.54-1.25 |
| Esophageal atresia |  |  |  |  |  |
| <15 minutes | 119 (51.5) | 1.00 |  | 1.00 |  |
| ≥15 minutes | 112 (48.5) | 0.86 | 0.66-1.12 | 0.98 | 0.72-1.32 |
| Gastroschisis |  |  |  |  |  |
| <15 minutes | 96 (25.8) | 1.00 |  | 1.00 |  |
| ≥15 minutes | 276 (74.2) | 2.63 | 2.07-3.35 | 1.46 | 1.10-1.94 |
| Omphalocele |  |  |  |  |  |
| <15 minutes | 63 (45.3) | 1.00 |  | 1.00 |  |
| ≥15 minutes | 76 (54.7) | 1.10 | 0.79-1.55 | 1.12 | 0.77-1.62 |
| Renal agenesis^d^ |  |  |  |  |  |
| <15 minutes | 23 (41.1) | 1.00 |  | 1.00 |  |
| ≥15 minutes | 33 (58.9) | 1.31 | 0.77-2.25 | 1.04 | 0.55-1.95 |
| Diaphragmatic hernia |  |  |  |  |  |
| <15 minutes | 124 (44.4) | 1.00 |  | 1.00 |  |
| ≥15 minutes | 155 (55.6) | 1.14 | 0.90-1.46 | 1.15 | 0.87-1.52 |
| Limb reduction defects |  |  |  |  |  |
| <15 minutes | 182 (47.8) | 1.00 |  | 1.00 |  |
| ≥15 minutes | 199 (52.2) | 1.00 | 0.81-1.24 | 0.99 | 0.78-1.26 |
| Hypospadias |  |  |  |  |  |
| <15 minutes | 405 (54.1) | 1.00 |  | 1.00 |  |
| ≥15 minutes | 344 (45.9) | 0.77 | 0.65-0.91 | 1.12 | 0.91-1.37 |

Abbreviations: OR, odds ratio; CI, confidence interval

^a^ Adjusted for surveillance center and maternal age at delivery, body mass index, education, race/ethnicity, income, parity, folic acid use, smoking, and season of conception

^c^ Includes anopthalmia

^d^ Bilateral renal agenesis or hypoplasia
